# Bacterial Symbionts Associated with Wild Tsetse Flies (Diptera: Glossinidae) in Sub-Saharan Africa: A Systematic Review and Meta-Analysis

**DOI:** 10.3390/life16071170

**Published:** 2026-07-15

**Authors:** Mmatlala Dorothy Nkosi, Moeti Oriel Taioe, ThankGod Emmanuel Onyiche, Ana Mbokeleng Tsotetsi-Khambule

**Affiliations:** 1 Department of Life and Consumer Sciences, College of Agriculture and Environmental Sciences, University of South Africa, Roodepoort 1709, South Africa; tkhamam@unisa.ac.za; 2 Agricultural Research Council—Onderstepoort Veterinary Institute, Soutpan Road (M35), Onderstepoort 0110, South Africa; taioem@arc.agric.za; 3 Unit for Environmental Sciences and Management, North-West University, Potchefstroom Campus, Private Bag X6001, Potchefstroom 2520, South Africa; et.onyiche@unimaid.edu.ng; 4 Department of Veterinary Parasitology and Entomology, University of Maiduguri, Maiduguri 1069, Nigeria

**Keywords:** *Glossina* spp., *Wigglesworthia*, *Sodalis*, *Wolbachia*, *Spiroplasma*, temporal trends

## Abstract

Bacterial symbionts play a significant role in the biology of tsetse flies (genus *Glossina*). However, their infection prevalence in *Glossina* spp. is not well documented. Hence, the systematic review followed PRISMA guidelines to determine bacterial infection prevalence among different *Glossina* spp. across sub-Saharan Africa. Six databases were used to search for studies published between 1980 and 2024. The search was conducted from February to March 2025. Meta-analysis was conducted using estimated pooled infection prevalence of bacterial symbionts in different *Glossina* species. Spearman’s rank correlation coefficient was calculated to examine changes in bacterial infection prevalence between 2010 and 2024. A total of 30 studies from 19 countries met the inclusion criteria and were included in the final systematic review. Of the 30 studies, 173 records were extracted consisting of data from 14 *Glossina* species. Burkina Faso (n = 25) recorded highest number of studies and *G. pallidipes* (n = 27) was the most studied species. Significant associations were observed between bacterial infection prevalence and the tested variables (*Glossina* spp. and country). Temporal analysis results showed positive association in *Spiroplasma* infection prevalence over the years (rho = 0.418, *p* = 0.017) whilst *Sodalis* infections showed negative association (rho = −0.319. *p* = 0.008). Heterogeneity among different studies was substantially high (I^2^ > 75%) and evidence of publication bias was reported. The results indicated that bacterial infection prevalence was influenced by factors such as country, sample size and symbiont-specific species. However, heterogeneity remained high even after meta-regression analysis suggesting that other variables not included in the analysis or rather the combination of different variables are responsible for the high heterogeneity observed. Despite study limitations, the study provided the most comprehensive and complex results on the relationship between bacterial symbiont infections and *Glossina* species.

## 1. Introduction

Tsetse flies (Diptera: Glossinidae) are economically and agriculturally blood-feeding insects endemic to sub-Saharan Africa [1,2]. The flies are cyclical vectors of *Trypanosoma* parasites that causes AAT (African Animal Trypanosomiasis) in animals and HAT (Human African Trypanosomiasis) in humans [1,3]. Tsetse flies belong to the genus *Glossina* which is divided into three subgenera, namely: riverine (*palpalis*), savannah (*morsitans*) and forest-dwelling [1,4]. The flies are known to carry bacterial symbionts as well as environmentally acquired bacteria [5]. Endosymbionts play a significant role in tsetse reproduction, feeding, metabolism and disease transmissions [6,7]. Earlier studies reported that tsetse flies harbor three maternally transmitted symbionts (*Wigglesworthia* spp., *Sodalis* spp. and *Wolbachia* spp.) [8,9,10]. However, recent studies have reported *Spiroplasma* as the fourth bacterial symbiont in tsetse flies [11,12,13,14].

The primary symbiont, *Wigglesworthia glossinidia* (Enterobacterales: Erwiniaceae) is found intracellularly in the bacteriocytes and extracellularly in the lumen of female milk glands [8,15,16]. This bacterium is transmitted maternally during intrauterine development and plays significant role in growth, development and the overall survival of tsetse flies [15]. The relationship between tsetse flies and *Wigglesworthia* spp. is mutualistic in which the bacterium provide B vitamins whilst in return, tsetse provide space and shelter for the bacterium [15,17,18]. Therefore, the absence of *Wigglesworthia* spp. in tsetse flies can lead to negative impacts on carbohydrates and amino acids metabolism, a compromised immune system in juvenile flies and sterility in adult females [18,19,20].

The secondary symbiont, *Sodalis glossinidius* (Enterobacterales: Pectobacteriaceae) is found intracellularly and extracellularly in different tissues including midgut, salivary glands, milk glands, body fats and hemocoel [17,21]. Similar to *Wigglesworthia*, *Sodalis* is also transmitted maternally via the milk secretion during intrauterine development [15]. The absence of *Sodalis* in tsetse flies is associated with reduced life span [22]. In addition, *Sodalis* is responsible for the establishment and maturation of trypanosome parasite in tsetse flies [23,24,25,26]. Furthermore, due to its cultivable abilities, *Sodalis* has been used in paratransgenesis approach studies to fight HAT in SSA [27,28,29].

The bacterium *Wolbachia* (Rickettsiales: Ehrlichiaceae) is found intracellularly in the germ line tissues of their host. Unlike *Wigglesworthia* and *Sodalis*, *Wolbachia* is transmitted transovarially through germ line cells [30]. Hence, the bacterium can be detected in oocytes, ovaries and testes of infected hosts [31,32,33]. *Wolbachia* has the ability to manipulate the reproductive biology of tsetse flies through mechanisms such as male killing, cytoplasmic incompatibility, parthenogenesis and feminization [34]. In addition, *Wolbachia* positive insects have high nutritional demand resulting in limited egg production [35].

*Spiroplasma* (Mycoplasmateles: Spiroplasmataceae) is another symbiont that is found intracellularly in different tissues and systematically in the haemolymph [36,37]. Studies on *Spiroplasma* in other insects reported that the bacterium acts as reproductive parasite that induces male-killing effects in some arthropods [38,39,40,41,42]. On the contrary, other studies reported that this bacterium offers protection against parasitoid wasps and nematodes in some *Drosophila* species [43,44]. In addition, given the medical importance of *Glossina* spp. as African trypanosomosis vectors, understanding their interaction with the host is important. Although, *Spiroplasma* has been extensively investigated in other insect species, studies on the prevalence of *Spiroplasma* in *Glossina* spp. only gained attention recently [12,13,45,46,47]. Therefore, the role of *Spiroplasma* in *Glossina* spp. is not well documented.

Understanding the bacterial infection prevalence in *Glossina* spp. is important for interpreting their role in vector biology [5,48,49]. Hence the aims of this review were to ascertain the bacterial symbionts associated with tsetse flies (Diptera: Glossinidae) and their pooled prevalence estimates across the sub-Saharan Africa using a systematic review and meta-analysis approach. The specific research questions that this review attempted to answer are: (i) Are there differences in infection prevalence between the bacterial symbionts? (ii) Are there differences between bacterial infection prevalence and *Glossina* spp. and between bacterial infection prevalence and country of host collection? (iii) Are there significant differences in pooled infection prevalence within *Glossina* spp.? (iv) How has the bacterial infection prevalence in tsetse flies changed over the year?

## 2. Materials and Methods

### 2.1. Search Strategy

For this review, the literature search was conducted in accordance with PRISMA (Preferred Reporting Items for Systematic reviews and Meta-Analysis) guidelines [50,51]. This systematic review was registered on OSF (Open Science Framework) under the ID: osf.io/nu7j3. Relevant studies were searched in the following electronic databases: Google scholar, PubMed, PubMed central, Science Direct, Scopus and Springer Link. Keywords used were tsetse fly OR *Glossina* OR bacterial symbionts OR tsetse flies’ bacterial symbionts OR *Glossina* bacterial symbionts. After removing duplicate records, all records of relevant titles and abstracts were screened by two authors (MDN and TEO) and articles that align with the aim of the study were downloaded and subjected to full-text screening based on the set of eligibility criteria.

### 2.2. Eligibility Criteria

The inclusion criteria for this systematic review were as follows: (i) the study collected wild tsetse flies within sub-Saharan Africa, (ii) identified the collected tsetse flies, (iii) screened the collected tsetse flies for bacterial symbionts, (iv) the country of study was included in the study, (v) stated the total number of tsetse flies screened, (vi) stated the exact number of tsetse flies positive to different bacterial symbiont, (vii) the availability of full-text articles, (viii) the method for the detection of bacterial symbiont isolation clearly stated, and (ix) studies published in English. Studies that failed to meet the above stated inclusion criteria were excluded.

### 2.3. Data Extraction and Analysis

All studies that met the inclusion criteria were saved on Mendeley reference manager according to their electronic database. A standardized data extraction form was designed in Microsoft Excel spreadsheet. All appropriate and relevant data were extracted and entered into the sheet. Data extracted on the Microsoft Excel sheet includes the following; author names, title, publication year, *Glossina* spp. bacterial symbiont, isolation method, country, sample size, infection prevalence, and reference. Additionally, cross-referencing of all included studies was conducted to check for more relevant studies. Microsoft Excel was further used to sort out the data. The open source QGIS version 3.38 was used to create the bacterial symbionts map included in the systematic review.

### 2.4. Assessment of Study Quality

The Joanna Briggs Institute (JBI) critical instrument for prevalence studies was used to assess the quality of the articles included in the systematic review [52]. The instrument consisted of nine “yes”, “no”, “unclear” and “not applicable” questions. All these questions were answered for each article, and the average score was calculated (Supplementary Materials).

### 2.5. Data Analysis

The IBM SPSS Statistics version 30 program (IBM Corp., Armonk, NY, USA) was used for comparative statistical analysis and the temporal trend analysis. Normality test was conducted using Kolmogorov–Smirnov and Shapiro–Wilk tests. Thereafter, Kruskal–Wallis non-parametric test was conducted to analyze the infection prevalence between different facultative bacterial symbionts. Similar test was conducted to check for the differences in bacterial infections and *Glossina* spp. and between countries of host collection.

Spearman’s rank correlation coefficient (rho) was calculated in SPSS to determine changes in each bacterial infection prevalence between 2010 and 2024. The bacterium *Wigglesworthia* was excluded from all the statistical comparative analysis, temporal trend analysis and meta-analysis due to its role as an obligate mutualistic bacterial symbiont of the tsetse flies.

The R-4.5.3 program was used for the meta-analysis and meta-regression. Each study was grouped based on the country of host collection, *Glossina* spp. analyzed, and bacteria. Sample size and positive cases were recorded for each study. The infection prevalence was calculated in R program. The prevalence proportions were log-transformed to stabilize variances and normalize distribution. The variance estimates were calculated for each study within the R environment using the Metafor package. For meta-analysis, random-effect model was fitted using GLMM (Generalized Linear Mixed Model). This method was used to account for study sampling error and between study heterogeneity. The method was also used because studies differ in geographical locations and *Glossina* species. The pooled prevalence estimates were back-transformed from the logit scale to proportion for interpretability. For heterogeneity assessment, Cochran’s Q-test was used to test for heterogeneity across studies. The I^2^ statistics quantified the proportion of total variability due to between study heterogeneity rather than chance. An I^2^ value greater than 75% was considered high. The T^2^ (Tau-squared) was used to estimate the difference between study variances. The publication bias was evaluated using Egger’s regression-based test and trim and fill method. The Egger’s regression-based test was conducted to assess the funnel plot asymmetry for publication bias. The trim and fill method was used to estimate the number of potentially missing studies and pooled prevalence estimates were adjusted accordingly.

Meta-regression analysis was conducted in R using the Metafor package. The GLMM was fitted and the logit-transformed proportion was used as the effect size measure. To explore the sources of heterogeneity, bacteria, country and sample size were used as moderators. The following *Glossina* spp. were excluded from the meta-analysis due to minimal number of studies; *G. brevipalpis*, *G. fuscipes quanzensis*, *G. medicorum*, *G. morsitans*, *G. morsitans centralis*, and *G. swynnertoni*.

## 3. Results

### 3.1. Study Selection

**Figure 1:**
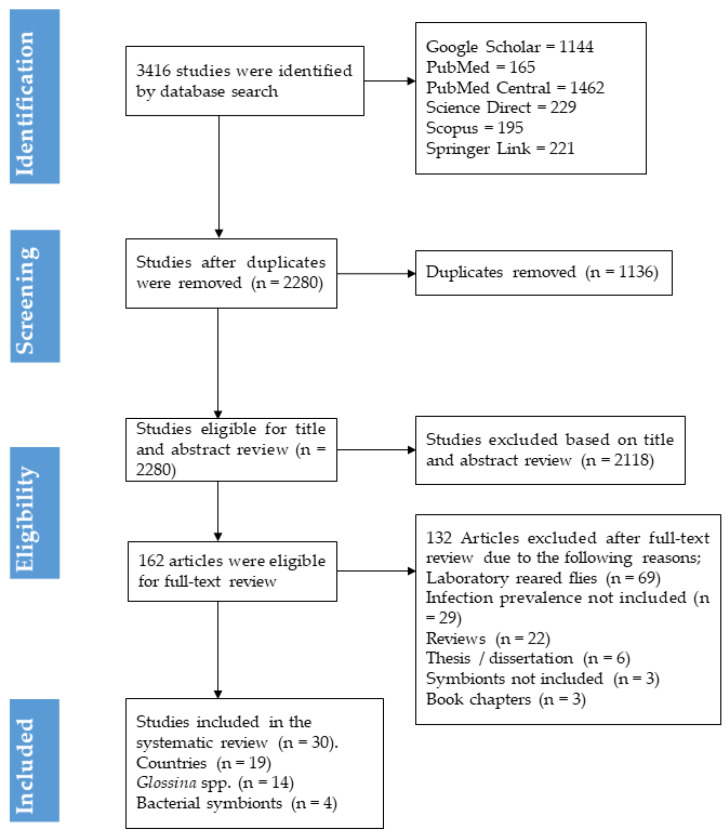
PRISMA flow chart of the selection process.

A total of 3416 published studies were identified through six databases. Of the total number of studies identified, 1136 were identified as duplicates and were removed. Thereafter, 2280 studies were eligible for title and abstract screening. A total of 2118 studies did not pass the title and abstract screening and were removed. These studies were mainly excluded due to the absence of bacterial symbionts or bacterial symbionts that were isolated from other insect species and not *Glossina* species. Of the remaining 162 studies that were eligible for full-text screening, 132 studies did not meet the inclusion criteria and were excluded from the study. Of the 132 studies excluded, 69 studies used laboratory reared tsetse flies, 29 studies did not include the infection prevalence, 22 studies were reviews, 6 studies were thesis/dissertations, 3 studies did not include tsetse bacterial symbionts, and 3 studies were book chapters. Therefore, only 30 articles were included in the final systematic review (Figure 1). These 30 articles were published between 2010 and 2024. Studies between 1980 and 2009 did not meet the inclusion criteria. The quality assessment score of the included studies using JBI critical appraisal ranged between 78% and 89%. A total of 26 studies scored 89% and 4 studies scored 78%. The four studies that recorded 78% were primarily due to limited samples size.

### 3.2. Descriptive Results

**Figure 2:**
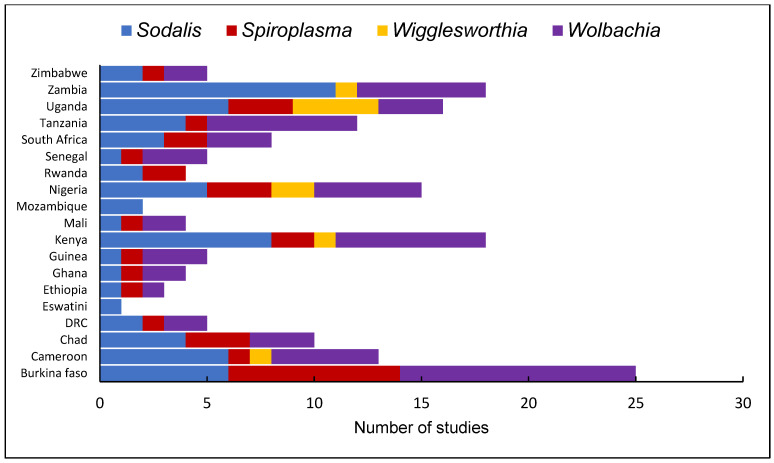
Number of studies recorded per country per bacterial symbiont.

Of the 30 reviewed articles, 173 records were extracted. Some studies isolated bacterial symbionts on more than one *Glossina* spp. and in more than one African country [11,53,54,55]. A total of 19 sub-Saharan African countries (Burkina Faso, Cameroon, Chad, Democratic Republic of Congo, Eswatini, Ethiopia, Guinea, Ghana, Kenya, Mali, Mozambique, Nigeria, Rwanda, Senegal, South Africa, Tanzania, Uganda, Zambia and Zimbabwe) conducted studies on the infection prevalence of bacterial symbionts from wild caught *Glossina* species (Appendix A, Table A1). Of the 173 extracted records, the highest number of studies were reported in Burkina Faso (n = 25) and the least number of studies was recorded in Eswatini (n = 1) (Figure 2).

Four bacterial symbionts (*Wigglesworthia*, *Sodalis*, *Wolbachia* and *Spiroplasma*) were isolated from 14 *Glossina* spp. (*G. austeni*, *G. brevipalpis*, *G. fuscipes fuscipes*, *G. fuscipes quanzensis*, *G. medicorum*, *G. morsitans centralis*, *G. morsitans*, *G. morsitans morsitans*, *G. morsitans submorsitans*, *G. palpalis gambiensis*, *G. palpalis palpalis*, *G. pallidipes*, *G. swynnertoni* and *G. tachinoides)* across the 19 sub-Saharan African countries (Appendix A, Table A1). The highest number of studies were recorded in *G. pallidipes* (n = 27).

### 3.3. Bacterial Infection Prevalence

**Figure 3:**
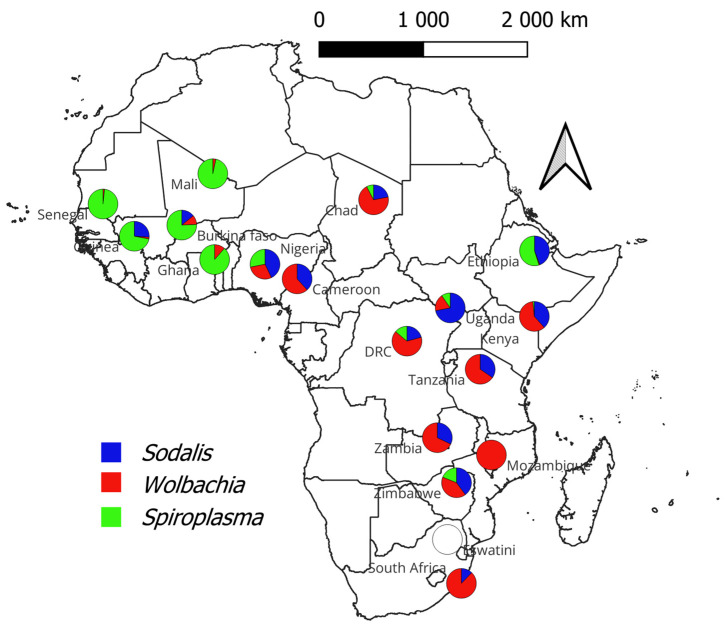
A map showing the infection prevalence of the bacterial symbionts in sub-Saharan Africa.

Four bacterial symbionts (*Wigglesworthia*, *Sodalis*, *Wolbachia* and *Spiroplasma*) were recorded across the 19 sub-Saharan African countries (Figure 3). The primary bacterium *Wigglesworthia* was reported in 5.2% (n = 9) of the total 173 records. *Sodalis* and *Wolbachia* accounted for 38.7% (n = 67) and 37.6% (n = 65) studies, respectively. The bacterium *Spiroplasma* was recorded in 18.5% (n = 32). The bacterium *Wigglesworthia* recorded 100% infection prevalence across all studies, whereas the infection prevalence in other facultative bacterial symbionts ranged between 0 and 100% (Appendix A, Table A1). The statistical analysis results using Kruskal–Wallis test showed that the infection prevalence did not differ significantly between the facultative bacterial symbionts (H = 0.146, df = 2, *p* = 0.930). On the contrary, significant differences were observed between facultative bacterial infection prevalence and country (H = 35.123, df = 18, *p* = 0.009). However, none of the 171 pairwise comparisons between countries was significant.

**Figure 4:**
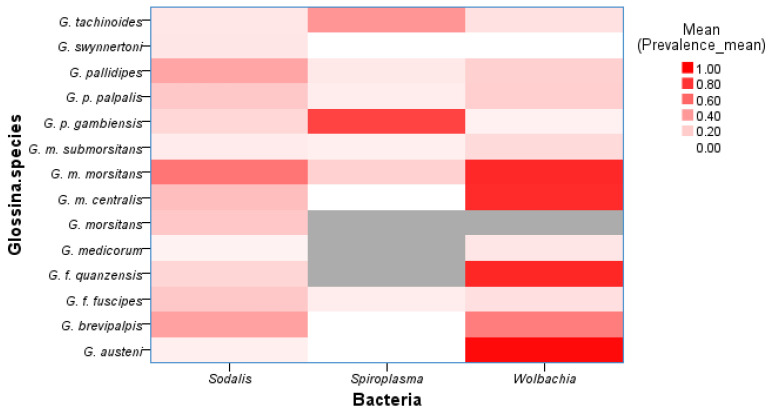
Heat map showing the mean infection prevalence between *Glossina* spp. and bacterial symbionts. The gray area represents no study records.

The heat map presented in Figure 4 showed that *Wolbachia* presented higher infection prevalence as compared to *Sodalis* and *Spiroplasma.* For example, High *Wolbachia* mean infection prevalence was observed in *G. m. morsitans*, *G. m. centralis*, *G. f. quanzensis* and *G. austeni*, whereas *G. p. gambiensis*, *G. m. centralis* and *G. medicorum* presented low *Wolbachia* infection prevalence. However, the overall mean infection prevalence across the three facultative bacterial symbionts prevalence varied according to *Glossina* species (Figure 4).

These visually observed results were supported statistically by Kruskal–Wallis test, which showed that facultative bacterial infection prevalence differed between *Glossina* spp. (H = 25,674, df = 13, *p* = 0.019). The 91 *Glossina* spp. pairwise comparisons results with Bonferroni adjustment showed that *G. m. morsitans* had significantly higher infection prevalence as compared to *G. m submorsitans* (*p* = 0.022), *G. p. gambiensis* (*p* = 0.050) and *G. tachinoides* (*p* = 0.010). All other remaining pairwise comparisons on *Glossina* spp. were non-significant.

### 3.4. Temporal Trends Analysis

**Figure 5:**
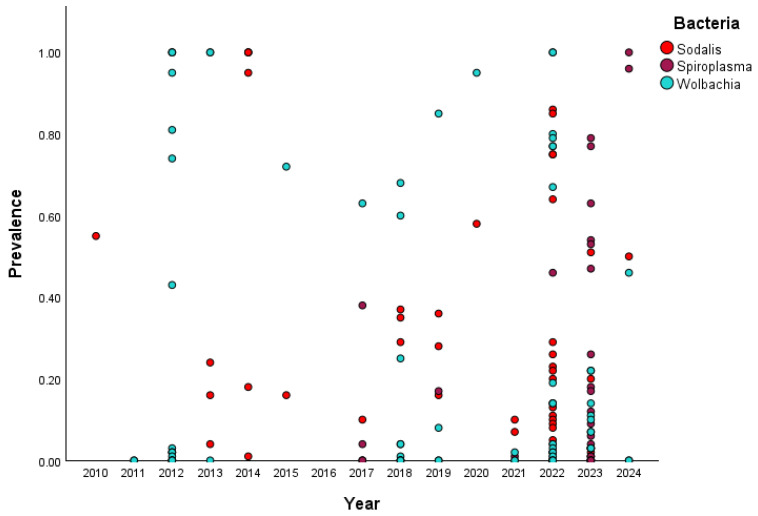
The scatter plot showing Spearman’s rand correlation results between 2010 and 2024.

Spearman’s rank correlation test between 2010 and 2024 indicated positive association between *Sodalis* infection prevalence and years (rho = −0.314, *p* = 0.0010). However, *Spiroplasma* infection prevalence showed negative association between study years (rho = 0.424, *p* = 0.016). Contrary, no significant trend was observed over the years with the bacterium *Wolbachia* (rho = 0.025, *p* = 0.845) indicating stable infection prevalence (Figure 5).

### 3.5. Pooling and Heterogeneity Analysis

**Figure 6:**
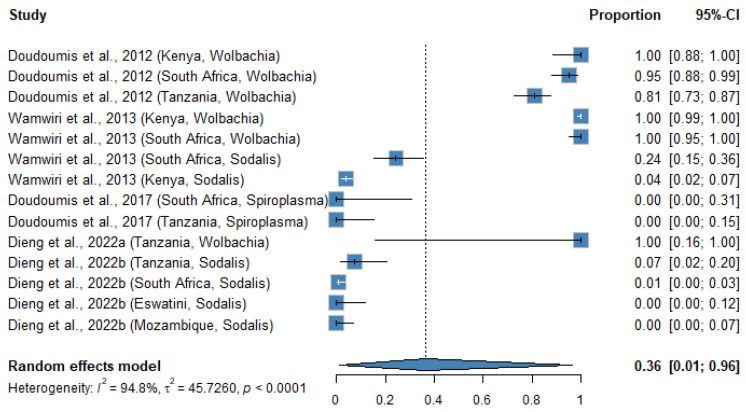
Forest plot for pooled infection prevalence in *G. austeni* [11,26,53,54,55].

*Glossina austeni:* A total of 14 studies were included in the meta-analysis for *G. austeni.* The meta-analysis results showed significantly pooled effect size. Additionally, high statistically significant heterogeneity was observed indicating that the variations in effect size across studies. Non-significant Egger’s regression-based test was recorded with zero missing studies indicating no evidence of publication bias (Table 1, Figure 6). The meta-regression results showed reduction in residual heterogeneity (T^2^ = 0.067, I^2^ = 54.62) and test for moderators was significant (QM = 86.16, *p* < 0.0001). Highly significant positive association was recorded with *Wolbachia* studies (β = 7.09, SE = 0.770, *p* < 0.0001, 95%CI [5.590, 8.607]) and significantly negative association (β = −2.772, SE = 0.864, CI [−4.466, −1.079], *p* = 0.0013) was recorded with samples size. On the contrary, country was not significant.

**Figure 7:**
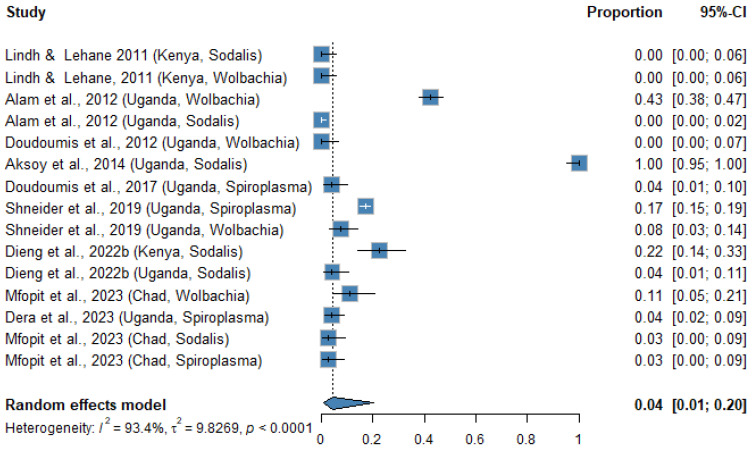
Forest plot for *G. f. fuscipes* [11,45,46,53,55,56,57,58,59].

*Glossina fuscipes fuscipes*: Fifteen studies were included in the meta-analysis. The significantly small, pooled effect size was observed and studies differed significantly with very high heterogeneity. The Egger’s regression-based test showed no publication bias (*p* = 0.06); however, the trim and fill suggest publication bias with 6 missing studies recorded (Table 1, Figure 7). The meta-regression results showed non-significant results in moderators (QM = 2.03, *p* = 0.845). In addition, heterogeneity remained significantly high (β = 0.207, 95% [0.072, 0.466], T^2^ = 8.72, I^2^ = 96.93%, Q = 316.17, *p* < 0.0001) and none of the tested moderators yielded significant results.

**Figure 8:**
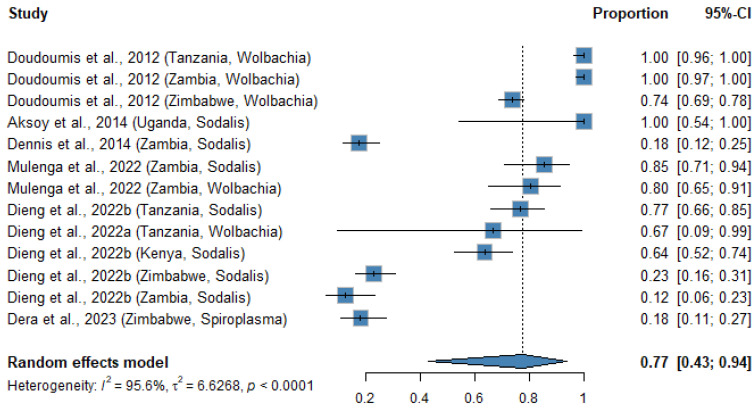
Forest plot for *G. m. morsitans* [45,53,54,55,58,60,61].

*Glossina morsitans morsitans*: A total of 13 studies were included in the meta-analysis and a strong statistically significant pooled effect was observed. A statistically high heterogeneity was also observed. Egger’s regression-based test was non-significant, however trim and fill method indicated publication bias with two missing studies. Evidence of publication bias was found with 2 missing studies (Table 1, Figure 8). The meta-regression results indicated significant results in moderators (QM = 19.78, *p* = 0.0061). Only the bacterium *Wolbachia* (β = 3.376, SE = 0.986, 95%CI [1.444, 5.309], *p* = 0.0005) is associated with high effect size. Other variables such as country and sample size were not associated with effect size and did not explain the high heterogeneity observed.

**Figure 9:**
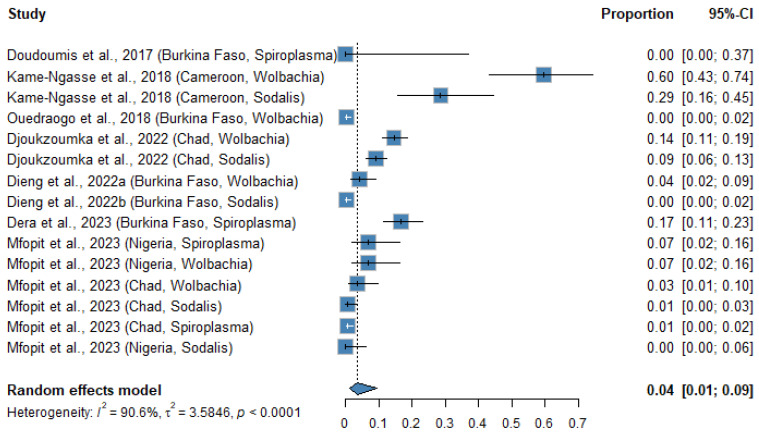
Forest plot for *G. m. submorsitans* [11,45,46,53,54,62,63,64].

*Glossina morsitans submorsitans*: Fifteen studies were included in the meta-analysis. The meta-analysis showed small but statistically significantly pooled effect. Additionally, high heterogeneity was observed. Strong evidence of publication bias was observed with 6 missing studies (Table 1, Figure 9). The meta-regression results showed non-significant results in moderators (QM = 2.87, *p* = 0.412). Heterogeneity remained significantly high (T^2^ = 2.77, I^2^ = 92.52%, Q = 115.32, *p* < 0.0001) and none of the tested moderators were significant.

**Figure 10:**
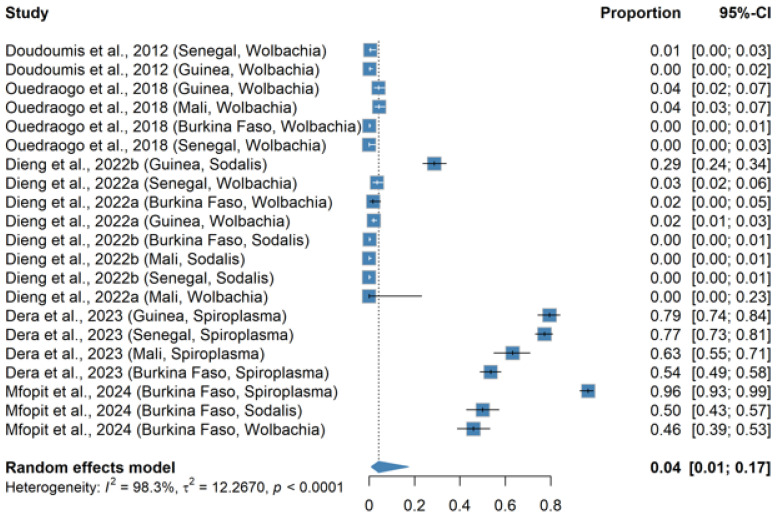
Forest plot for *G. p. gambiensis* [45,53,54,55,63,65].

*Glossina palpalis gambiensis*: A total of 21 studies were included in the meta-analysis. Meta-analysis results showed a small but statistically significant pooled effect size. Extremely high heterogeneity was observed among studies. Egger’s regression-based test showed strong evidence of publication bias was found with 9 studies missing (Table 1, Figure 10). The meta-regression results showed significant results in moderators (QM = 36.79, *p* < 0.0001). *Spiroplasma* (β = 5.348, SE = 1.302, 95%CI [2.796, 7.892], *p* < 0.0001) is significantly associated with higher effect size whilst Country, sample size and other bacterial symbionts were non-significant. Residual heterogeneity remained significantly high (T^2^ = 3.5, I^2^ = 97.36%).

**Figure 11:**
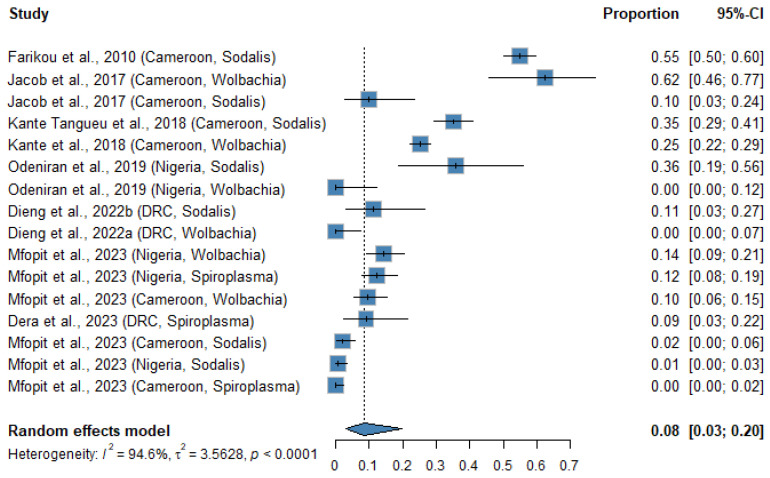
Forest plot for *G. p. palpalis* [24,45,46,53,54,66,67,68,69].

*Glossina palpalis palpalis:* Sixteen studies were included in the meta-analysis and showed small to moderate statistically significant positive effect size. Statistically significant heterogeneity was observed. The Egger’s regression-based test showed evidence of publication bias with 7 studies missing (Table 1, Figure 11). The test for moderators was non-significant (QM = 2.20, *p* = 0.822). None of the tested variables were significant with meta-regression analysis.

**Figure 12:**
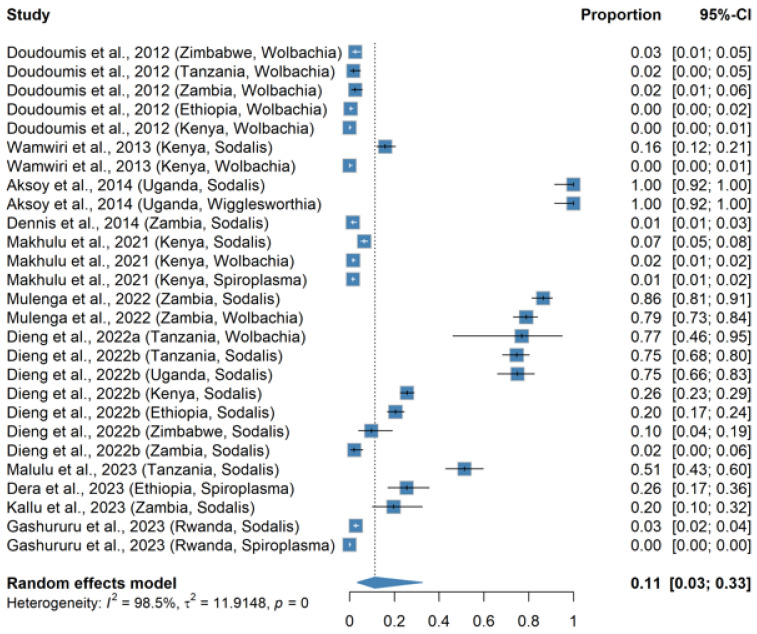
Forest plot for *G. pallidipes* [13,26,45,53,54,55,58,60,61,70,71,72].

*Glossina pallidipes*: Twenty-six studies were included in the meta-analysis for *G. pallidipes.* Small but statistically significant positive effect size was observed with extremely high heterogeneity among studies. No evidence of publication bias observed with Egger’s regression-based test; however, trim and fill method showed 7 missing studies (Table 1, Figure 12). Meta-regression results showed that test for moderators was significant (Q = 32.06, *p* = 0.0002) with bacterium *Wolbachia* (β = −2.322, SE = 0.902, 95%CI [−4.119, −0.586], *p* = 0.0091) showing significant effect on the effect size. Other bacterial species, country and sampling sites were not significant and heterogeneity still remained very high (T^2^ = 3.69, I^2^ = 98.45).

**Figure 13:**
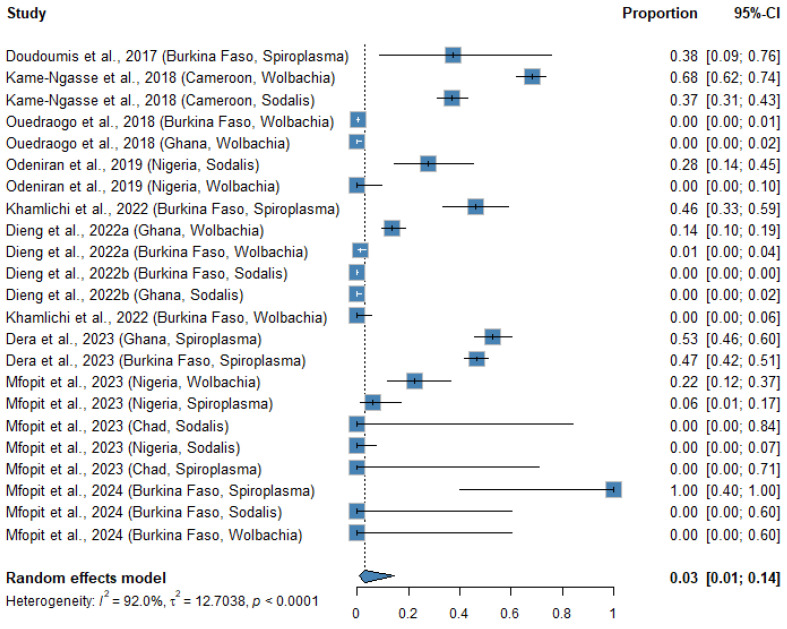
Forest plot for *G. tachinoides* [11,12,45,46,53,54,62,63,65,69].

*Glossina tachinoides*: A total of 24 studies were included in the meta-analysis and a small but significant positive effect size was observed. Significantly high heterogeneity observed. Egger’s regression-based test showed evidence of publication bias among *G. tachinoides* studies with 10 missing studies (Table 1, Figure 13). Test for moderators was significant (QM = 23.36, *p* = 0.0002), however residual heterogeneity still remained very high (T^2^ = 3.61, I^2^ = 93.02). Meta-regression analysis showed that the bacterium *Wolbachia* (β = 5.466, SE = 1.437, 95%CI [2.648, 8.283], *p* = 0.001) and Cameroon (β = 6.332, SE = 1.629, 95%CI [3.134, 9.524], *p* = 0.001) were significant whilst the remaining variables were non-significant.

### 3.6. Publication Bias

Evidence of publication bias was recorded in all studies except *G. austeni* and *G. pallidipes* studies (Table 1).

## 4. Discussion

The beneficial symbiosis with maternally transmitted bacteria is common in many insect species including tsetse flies [14,17,20,56,57,58,59]. These bacteria play different roles in the host’s biological processes such as feeding, metabolism and reproduction [6,7,17,60,61]. However, some of these bacteria rather cause harm to their hosts by compromising their immune and reproductive system and further transmitting different pathogens [30,36,39,62,63].

The current systematic review and meta-analysis provided an overview on the prevalence of bacterial symbionts isolated from different *Glossina* spp. across different sub-Saharan African countries. The comparative analysis results showed non-significant association between facultative bacterial symbionts and the infection prevalence. These results are supported by various previous experimental studies that recorded varying infection prevalence of facultative bacterial symbionts in wild-caught tsetse flies [11,31,46,55]. Thereafter, a heat map was created to visualize the infection prevalence across the three facultative bacterial symbionts. Although, the statistical analysis showed non-significant results between infection prevalence and bacterial symbionts, the heat map results showed that *Wolbachia* has high infection prevalence as compared to *Sodalis* and *Spiroplasma.* These results correlate with other studies that showed that *Wolbachia* is more prevalent than *Sodalis* in natural populations of tsetse flies [26,55].

Moreover, the infection prevalence differed significantly with country of host collection. However, no significant results were observed for pairwise comparisons. These results suggest that the three facultative bacterial symbiont infection prevalence in tsetse flies is not shaped by geographical areas only but rather by the combination of other different factors such as vegetation, temperature, humidity, *Glossina* spp. genetic structure and host feeding choices [26,46,64,65,66]. The results further showed that *Glossina* spp. is a significant regulator for the bacterial symbiont infection prevalence; however, pairwise comparisons only showed that *G. m. morsitans* had significantly higher infection prevalence as compared to *G. m. submorsitans*, *G. p. gambiensis* and *G. tachinoides.* These results suggest that symbiont infection prevalence in tsetse flies are species-specific. Notably, tsetse flies have different genetic structure composition which influence the establishment of symbionts, genotype by symbionts specificity, immune gene polymorphism, tissue distribution, population density and vector competence. Therefore, the observed significant infection prevalence in *Glossina* spp. was likely driven by a combination of different factors such as environmental conditions, host immune system, host genetic variations, microbe-microbe interaction and host blood meal sources [2,13,22,67,68,69].

Spearman’s rank correlation test for temporal trends showed no significant differences in *Wolbachia* infection prevalence over the years. However, *Sodalis* showed significantly negative association whilst *Spiroplasma* showed significantly positive association over the years. While *Wolbachia* showed stability on the temporal trends analysis results, *Sodalis* and *Spiroplasma* showed significant changes. The results observed in *Sodalis* and *Spiroplasma* suggest that the bacteria symbionts responds to environmental conditions and changes [70]. The low *Sodalis* infection prevalence observed on the heat map coupled with significant negative association over the years indicate an unstable relationship with their host and non-tolerance to environmental changes [46]. In addition, *Sodalis* infections were found to decrease with increase age in *G. pallidipes* [62]. The temporal analysis results further showed positive association in *Spiroplasma* infection prevalence over the years indicating that the infection prevalence vary greatly among different *Glossina* species [11,71,72]. The variations in *Spiroplasma* infection could be influenced by factors such as sampling location, environmental conditions and the overall host biology [71].

Significantly high heterogeneity (I^2^ > 75%) was observed across all analyzed *Glossina* spp. studies. Bacterial symbionts were used as subgroups and showed significant pooled infection prevalence that is dependent of *Glossina* species. Furthermore, Meta-regression analysis results showed that bacteria, sample size and country drove heterogeneity across studies. However, the bacterium *Wolbachia* seemed to stand out because it was the most significant bacteria in the meta-analysis as compared to *Sodalis* and *Spiroplasma.* Although, some moderators were significant in some *Glossina* spp. studies, they failed to explain the high heterogeneity observed across the studies. These results suggest that some other unmeasured biological variables and methodology are responsible for the high heterogeneity observed [73,74]. The publication bias methods used suggested potential bias in all *Glossina* spp. related studies except in *G. austeni* and *G. pallidipes* studies indicating that statistical power was influenced due to small study-effects; therefore, the results must be interpreted cautiously.

Another factor to discuss is the domination of bacterium *Wolbachia* in the whole study as compared to *Sodalis* and *Spiroplasma. Wolbachia* has shown to have high infection prevalence on the heat map, stable with the temporal trend analysis and was further significant in several *Glossina* spp. studies with the meta-regression analysis. The consistent prevalent and temporal trends stability may reflect efficient transmission dynamics. *Wolbachia* uses reproductive manipulation strategy such as cytoplasmic incompatibility to populate itself into host population [34,55]. Additionally, the bacterium is transmitted transovarially which ensures high transmission efficiency and is less sensitive to environmental conditions [31,32,33]. On the contrary, *Sodalis* lacks aggressive reproductive drive strategy as compared to *Wolbachia* and is more sensitive to environmental fluctuation [70]. Similar to *Sodalis*, the bacterium *Spiroplasma* is also sensitive to environmental changes and is known to compete with the host for nutrients which can lead to delayed larval development and compromised sperm fitness in male tsetse flies [47].

While the study provided a comprehensive overview on the prevalence of bacterial symbionts isolated from different *Glossina* spp., there were limitations that must be considered when interpreting the results from this study. Some *Glossina* spp. (*G. brevipalpis*, *G. medicorum*, *G. morsitans*, *G. m. centralis*, *G. quanzensis*, and *G. swynnertoni*) had limited number of studies and could not be included in the meta-analysis. In addition, small samples size in some studies could have affected the accuracy and the reliabilities of some estimates. This is supported by the JBI critical appraisal results. Although the overall quality of the included studies was high, inadequate sample size was identified in a subset of studies. Many studies emphasized that smaller sample size can lead to wider confidence level and lower precision in prevalence studies and does not portray the true picture of the infected individuals in a population [75,76]. Although JBI critical appraisal tool was conducted to check for the quality of the studies, the effects of within-study quality differences on the infection prevalence estimates were not conducted. The inclusion of only English-language-based studies could have been a limiting factor and introduced language bias in the study. The detection method across studies was not included in the study and may have contributed to infection prevalence and the heterogeneity results observed. Lastly, the sensitivity analysis was not conducted which could have limited the ability to assess the robustness of the pooled estimates. However, despite these limitations, the study still provided an insight into infection prevalence among bacterial symbionts, complex relationship between wild-caught *Glossina* spp. and bacterial symbionts infections as well as the temporal trends in bacterial symbionts across different *Glossina* species.

## 5. Conclusions

The systematic review and meta-analysis provided the most comprehensive results to date on the bacterial symbionts in *Glossina* spp. across the sub-Saharan Africa. The bacterial infection prevalence in tsetse flies vary significantly among *Glossina* spp. and country of host collection. The bacterium *Wolbachia* exhibited higher infection prevalence, temporal stability and frequent significance in the meta-regression analysis indicating that *Wolbachia* represent stable, widespread and efficient transmission dynamics. The high heterogeneity observed across different *Glossina* spp. are influenced by rather complex variables that were not included in the analysis. Furthermore, the study highlighted the complex relationship between bacterial infection prevalence and different tsetse species.

## Figures and Tables

**Table 1:** Meta-analysis of the pooled infection prevalence estimates across different *Glossina* spp.

*Glossina* spp.	Total Studies	Pooled Prevalence		Test for Heterogeneity				Publication Bias	
		Effect Size	95%CI	T^2^	I^2^	Q Value	Q *p*-Value	Egger’s *p*-Value	Missing Studies
*G. austeni*	14	0.365	0.012, 0.965	13.19	94.8	248.35	<0.0001	0.891	0
*G. f. fuscipes*	15	0.043	0.008, 0.201	9.83	93.4	212.52	<0.0001	0.064	6
*G. m. morsitans*	13	0.773	0.431, 0.939	6.63	95.6	270.79	<0.0001	0.833	2
*G. m. submorsitans*	15	0.035	0.013, 0.094	3.58	91	149.57	<0.0001	0.035	6
*G. p. gambiensis*	21	0.042	0.009, 0.174	12.28	98.3	1167.38	<0.0001	0.0036	9
*G. p. palpalis*	16	0.084	0.033, 0.199	3.56	94.8	278.24	<0.0001	0.019	7
*G. pallidipes*	26	0.089	0.028, 0.252	11.91	98.5	1709.24	0	0.196	0
*G. tachinoides*	23	0.029	0.005, 0.144	12.70	92	275.73	<0.0001	0.0013	1

## Data Availability

The original contributions presented in this study are included in the article/Supplementary Material. Further inquiries can be directed to the corresponding author.

## Supplementary Materials

The following supporting information can be downloaded at https://www.mdpi.com/article/10.3390/life16071170/s1, Supplementary File S1: Quality assessment scores for eligible studies.

## Abbreviations

The following abbreviations are used in this manuscript:

DRC	Democratic Republic of Congo
GLMM	Generalized Linear Mixed Model
OSF	Open Science Framework
PRISMA	Preferred Reporting Items for Systematic Reviews and Meta-Analysis
QGIS	Quantum Geographic Information System
SSA	Sub-Saharan Africa

## Appendix A

**Table A1:** General characteristics of all studies included in the systematic review showing the association of bacterial symbionts with different *Glossina* spp. across sub-Saharan Africa.

Bacteria	*Glossina* spp.	Country	Sample Size	Positive Cases	Infection Prevalence	References
*Sodalis*	*G. austeni*	South Africa	74	18	24.32	[26]
*Sodalis*	*G. austeni*	Kenya	296	11	3.72	[26]
*Sodalis*	*G. austeni*	Tanzania	40	3	7.5	[53]
*Sodalis*	*G. austeni*	South Africa	226	2	0.88	[53]
*Sodalis*	*G. austeni*	Eswatini	30	0	0	[53]
*Sodalis*	*G. austeni*	Mozambique	50	0	0	[53]
*Sodalis*	*G. brevipalpis*	Zambia	55	52	94.55	[63]
*Sodalis*	*G. brevipalpis*	Mozambique	50	7	14	[53]
*Sodalis*	*G. brevipalpis*	South Africa	300	7	2.33	[53]
*Sodalis*	*G. f. fuscipes*	Kenya	64	0	0	[77]
*Sodalis*	*G. f. fuscipes*	Uganda	222	0	0	[78]
*Sodalis*	*G. f. fuscipes*	Uganda	76	76	100	[79]
*Sodalis*	*G. f. fuscipes*	Kenya	89	20	22.47	[53]
*Sodalis*	*G. f. fuscipes*	Uganda	94	4	4.26	[53]
*Sodalis*	*G. f. fuscipes*	Chad	75	2	2.67	[46]
*Sodalis*	*G. f. quanzensis*	DRC	360	56	15.56	[80]
*Sodalis*	*G. m. centralis*	Zambia	69	11	15.94	[81]
*Sodalis*	*G. m. centralis*	Zambia	85	49	57.65	[10]
*Sodalis*	*G. m. centralis*	Rwanda	330	10	3	[82]
*Sodalis*	*G. m. morsitans*	Uganda	6	6	100	[79]
*Sodalis*	*G. m. morsitans*	Zambia	137	24	17.52	[63]
*Sodalis*	*G. m. morsitans*	Zambia	41	35	85.37	[65]
*Sodalis*	*G. m. morsitans*	Tanzania	81	62	76.54	[53]
*Sodalis*	*G. m. morsitans*	Kenya	85	54	63.53	[53]
*Sodalis*	*G. m. morsitans*	Zimbabwe	139	32	23.02	[53]
*Sodalis*	*G. m. morsitans*	Zambia	64	8	12.5	[53]
*Sodalis*	*G. m. submorsitans*	Cameroon	42	12	28.57	[83]
*Sodalis*	*G. m. submorsitans*	Chad	345	31	8.99	[84]
*Sodalis*	*G. m. submorsitans*	Burkina Faso	343	1	0.29	[53]
*Sodalis*	*G. m. submorsitans*	Chad	182	1	0.55	[46]
*Sodalis*	*G. m. submorsitans*	Nigeria	60	0	0	[46]
*Sodalis*	*G. medicorum*	Burkina Faso	154	8	5.19	[53]
*Sodalis*	*G. morsitans*	Zambia	270	60	22.22	[85]
*Sodalis*	*G. p. gambiensis*	Guinea	314	90	28.66	[53]
*Sodalis*	*G. p. gambiensis*	Burkina Faso	943	2	0.21	[53]
*Sodalis*	*G. p. gambiensis*	Mali	364	0	0	[53]
*Sodalis*	*G. p. gambiensis*	Senegal	547	0	0	[53]
*Sodalis*	*G. p. gambiensis*	Burkina Faso	196	98	50	[72]
*Sodalis*	*G. p. palpalis*	Cameroon	450	247	54.89	[24]
*Sodalis*	*G. p. palpalis*	Cameroon	40	4	10	[58]
*Sodalis*	*G. p. palpalis*	Cameroon	274	96	35.04	[86]
*Sodalis*	*G. p. palpalis*	Nigeria	28	10	35.71	[87]
*Sodalis*	*G. p. palpalis*	DRC	35	4	11.43	[53]
*Sodalis*	*G. p. palpalis*	Cameroon	149	3	2.01	[46]
*Sodalis*	*G. p. palpalis*	Nigeria	161	1	0.62	[46]
*Sodalis*	*G. pallidipes*	Kenya	302	48	15.89	[26]
*Sodalis*	*G. pallidipes*	Uganda	42	42	100	[79]
*Sodalis*	*G. pallidipes*	Zambia	419	6	1.43	[63]
*Sodalis*	*G. pallidipes*	Kenya	1136	74	6.51	[13]
*Sodalis*	*G. pallidipes*	Zambia	237	205	86.5	[65]
*Sodalis*	*G. pallidipes*	Tanzania	217	162	74.65	[53]
*Sodalis*	*G. pallidipes*	Uganda	116	87	75	[53]
*Sodalis*	*G. pallidipes*	Kenya	834	214	25.66	[53]
*Sodalis*	*G. pallidipes*	Ethiopia	459	94	20.48	[53]
*Sodalis*	*G. pallidipes*	Zimbabwe	72	7	9.72	[53]
*Sodalis*	*G. pallidipes*	Zambia	146	3	2.05	[53]
*Sodalis*	*G. pallidipes*	Tanzania	146	75	51.37	[88]
*Sodalis*	*G. pallidipes*	Zambia	56	11	19.64	[85]
*Sodalis*	*G. pallidipes*	Rwanda	771	21	2.7	[82]
*Sodalis*	*G. swynnertoni*	Kenya	31	3	9.68	[13]
*Sodalis*	*G. tachinoides*	Cameroon	270	100	37.04	[83]
*Sodalis*	*G. tachinoides*	Nigeria	36	10	27.78	[87]
*Sodalis*	*G. tachinoides*	Burkina Faso	834	0	0	[53]
*Sodalis*	*G. tachinoides*	Ghana	234	0	0	[53]
*Sodalis*	*G. tachinoides*	Chad	2	0	0	[46]
*Sodalis*	*G. tachinoides*	Nigeria	49	0	0	[46]
*Sodalis*	*G. tachinoides*	Burkina Faso	4	0	0	[72]
*Spiroplasma*	*G. austeni*	South Africa	10	0	0	[11]
*Spiroplasma*	*G. austeni*	Tanzania	22	0	0	[11]
*Spiroplasma*	*G. brevipalpis*	South Africa	230	0	0	[45]
*Spiroplasma*	*G. f. fuscipes*	Uganda	98	4	4.08	[11]
*Spiroplasma*	*G. f. fuscipes*	Uganda	1415	243	17.17	[71]
*Spiroplasma*	*G. f. fuscipes*	Uganda	147	6	4.08	[45]
*Spiroplasma*	*G. f. fuscipes*	Chad	77	2	2.6	[46]
*Spiroplasma*	*G. m. centralis*	Rwanda	330	0	0	[82]
*Spiroplasma*	*G. m. morsitans*	Zimbabwe	94	17	18.09	[45]
*Spiroplasma*	*G. m. submorsitans*	Burkina Faso	8	0	0	[11]
*Spiroplasma*	*G. m. submorsitans*	Burkina Faso	169	28	16.57	[45]
*Spiroplasma*	*G. m. submorsitans*	Nigeria	60	4	6.67	[46]
*Spiroplasma*	*G. m. submorsitans*	Chad	343	2	0.58	[46]
*Spiroplasma*	*G. p. gambiensis*	Guinea	282	224	79.43	[45]
*Spiroplasma*	*G. p. gambiensis*	Senegal	491	379	77.19	[45]
*Spiroplasma*	*G. p. gambiensis*	Mali	152	96	63.16	[45]
*Spiroplasma*	*G. p. gambiensis*	Burkina Faso	471	252	53.5	[45]
*Spiroplasma*	*G. p. gambiensis*	Burkina Faso	196	189	96.43	[72]
*Spiroplasma*	*G. p. palpalis*	Nigeria	161	20	12.42	[72]
*Spiroplasma*	*G. p. palpalis*	DRC	44	4	9.09	[45]
*Spiroplasma*	*G. p. palpalis*	Cameroon	154	0	0	[46]
*Spiroplasma*	*G. pallidipes*	Kenya	1136	17	1.5	[13]
*Spiroplasma*	*G. pallidipes*	Ethiopia	94	24	25.53	[45]
*Spiroplasma*	*G. pallidipes*	Rwanda	771	0	0	[82]
*Spiroplasma*	*G. swynnertoni*	Kenya	31	0	0	[13]
*Spiroplasma*	*G. tachinoides*	Burkina Faso	8	3	37.5	[11]
*Spiroplasma*	*G. tachinoides*	Burkina Faso	63	29	46.03	[12]
*Spiroplasma*	*G. tachinoides*	Ghana	187	99	52.94	[45]
*Spiroplasma*	*G. tachinoides*	Burkina Faso	466	217	46.57	[45]
*Spiroplasma*	*G. tachinoides*	Nigeria	49	3	6.12	[46]
*Spiroplasma*	*G. tachinoides*	Chad	3	0	0	[46]
*Spiroplasma*	*G. tachinoides*	Burkina Faso	4	4	100	[72]
*Wigglesworthia*	*G. f. fuscipes*	Kenya	64	64	100	[77]
*Wigglesworthia*	*G. f. fuscipes*	Uganda	477	477	100	[78]
*Wigglesworthia*	*G. f. fuscipes*	Uganda	76	76	100	[79]
*Wigglesworthia*	*G. m. centralis*	Zambia	85	85	100	[10]
*Wigglesworthia*	*G. m. morsitans*	Uganda	6	6	100	[79]
*Wigglesworthia*	*G. p. palpalis*	Cameroon	40	40	100	[58]
*Wigglesworthia*	*G. p. palpalis*	Nigeria	28	28	100	[87]
*Wigglesworthia*	*G. pallidipes*	Uganda	42	42	100	[79]
*Wigglesworthia*	*G. tachinoides*	Nigeria	36	28	77.78	[87]
*Wolbachia*	*G. austeni*	Kenya	30	30	100	[55]
*Wolbachia*	*G. austeni*	South Africa	83	79	95.18	[55]
*Wolbachia*	*G. austeni*	Tanzania	120	97	80.83	[55]
*Wolbachia*	*G. austeni*	Kenya	296	296	100	[26]
*Wolbachia*	*G. austeni*	South Africa	74	74	100	[26]
*Wolbachia*	*G. austeni*	Tanzania	2	2	100	[54]
*Wolbachia*	*G. brevipalpis*	South Africa	50	1	2	[55]
*Wolbachia*	*G. brevipalpis*	Tanzania	2	2	100	[54]
*Wolbachia*	*G. f. fuscipes*	Kenya	64	0	0	[77]
*Wolbachia*	*G. f. fuscipes*	Uganda	477	203	42.56	[78]
*Wolbachia*	*G. f. fuscipes*	Uganda	53	0	0	[55]
*Wolbachia*	*G. f. fuscipes*	Uganda	106	8	7.55	[71]
*Wolbachia*	*G. f. fuscipes*	Chad	71	8	11.27	[46]
*Wolbachia*	*G. f. quanzensis*	DRC	184	156	84.78	[80]
*Wolbachia*	*G. m. centralis*	Zambia	69	50	72.46	[81]
*Wolbachia*	*G. m. centralis*	Zambia	85	81	95.29	[10]
*Wolbachia*	*G. m. morsitans*	Tanzania	100	100	100	[55]
*Wolbachia*	*G. m. morsitans*	Zambia	122	122	100	[55]
*Wolbachia*	*G. m. morsitans*	Zimbabwe	379	279	73.61	[55]
*Wolbachia*	*G. m. morsitans*	Zambia	41	33	80.49	[65]
*Wolbachia*	*G. m. morsitans*	Tanzania	3	2	66.67	[54]
*Wolbachia*	*G. m. submorsitans*	Cameroon	42	25	59.52	[83]
*Wolbachia*	*G. m. submorsitans*	Burkina Faso	343	1	0.29	[89]
*Wolbachia*	*G. m. submorsitans*	Chad	345	50	14.49	[84]
*Wolbachia*	*G. m. submorsitans*	Burkina Faso	142	6	4.23	[54]
*Wolbachia*	*G. m. submorsitans*	Nigeria	60	4	6.67	[46]
*Wolbachia*	*G. m. submorsitans*	Chad	86	3	3.49	[46]
*Wolbachia*	*G. medicorum*	Burkina Faso	154	1	0.65	[89]
*Wolbachia*	*G. medicorum*	Burkina Faso	94	18	19.15	[54]
*Wolbachia*	*G. p. gambiensis*	Senegal	188	1	0.53	[55]
*Wolbachia*	*G. p. gambiensis*	Guinea	367	1	0.27	[55]
*Wolbachia*	*G. p. gambiensis*	Guinea	314	13	4.14	[89]
*Wolbachia*	*G. p. gambiensis*	Mali	364	16	4.4	[89]
*Wolbachia*	*G. p. gambiensis*	Burkina Faso	731	1	0.14	[89]
*Wolbachia*	*G. p. gambiensis*	Senegal	128	0	0	[89]
*Wolbachia*	*G. p. gambiensis*	Senegal	316	11	3.48	[54]
*Wolbachia*	*G. p. gambiensis*	Burkina Faso	171	3	1.75	[54]
*Wolbachia*	*G. p. gambiensis*	Guinea	693	14	2.02	[54]
*Wolbachia*	*G. p. gambiensis*	Mali	14	0	0	[54]
*Wolbachia*	*G. p. gambiensis*	Burkina Faso	196	90	45.92	[72]
*Wolbachia*	*G. p. palpalis*	Cameroon	40	25	62.5	[58]
*Wolbachia*	*G. p. palpalis*	Cameroon	790	200	25.32	[90]
*Wolbachia*	*G. p. palpalis*	Nigeria	28	0	0	[87]
*Wolbachia*	*G. p. palpalis*	DRC	48	0	0	[54]
*Wolbachia*	*G. p. palpalis*	Nigeria	161	23	14.29	[46]
*Wolbachia*	*G. p. palpalis*	Cameroon	155	15	9.68	[46]
*Wolbachia*	*G. pallidipes*	Zimbabwe	355	9	2.54	[55]
*Wolbachia*	*G. pallidipes*	Tanzania	177	3	1.69	[55]
*Wolbachia*	*G. pallidipes*	Zambia	203	5	2.46	[55]
*Wolbachia*	*G. pallidipes*	Ethiopia	454	2	0.44	[55]
*Wolbachia*	*G. pallidipes*	Kenya	470	0	0	[55]
*Wolbachia*	*G. pallidipes*	Kenya	302	0	0	[26]
*Wolbachia*	*G. pallidipes*	Kenya	1136	18	1.58	[13]
*Wolbachia*	*G. pallidipes*	Zambia	237	187	78.9	[65]
*Wolbachia*	*G. pallidipes*	Tanzania	13	10	76.92	[54]
*Wolbachia*	*G. swynnertoni*	Kenya	31	0	0	[13]
*Wolbachia*	*G. tachinoides*	Cameroon	270	184	68.15	[83]
*Wolbachia*	*G. tachinoides*	Burkina Faso	834	2	0.24	[89]
*Wolbachia*	*G. tachinoides*	Ghana	234	0	0	[89]
*Wolbachia*	*G. tachinoides*	Nigeria	36	0	0	[87]
*Wolbachia*	*G. tachinoides*	Ghana	234	32	13.68	[54]
*Wolbachia*	*G. tachinoides*	Burkina Faso	199	2	1.01	[54]
*Wolbachia*	*G. tachinoides*	Burkina Faso	63	0	0	[12]
*Wolbachia*	*G. tachinoides*	Nigeria	49	11	22.45	[46]
*Wolbachia*	*G. tachinoides*	Burkina Faso	4	0	0	[72]
